# Food seeking in spite of harmful consequences is under prefrontal cortical noradrenergic control

**DOI:** 10.1186/1471-2202-11-15

**Published:** 2010-02-08

**Authors:** Emanuele Claudio Latagliata, Enrico Patrono, Stefano Puglisi-Allegra, Rossella Ventura

**Affiliations:** 1 Santa Lucia Foundation, European Centre for Brain Research (CERC), via del Fosso di Fiorano 64, Rome, 00143 Italy; 2 Dipartimento di Psicologia and Centro "Daniel Bovet", University "La Sapienza", piazzale Aldo Moro 5 Rome, 00181 Italy; 3 Dipartimento di Scienze e Tecnologie Biomediche, University of L'Aquila, via Vetoio (Coppito 2) Coppito, L'Aquila, Italy 67010 Italy

## Abstract

**Background:**

Eating disorders are multifactorial psychiatric disorders. Chronic stressful experiences and caloric restriction are the most powerful triggers of eating disorders in human and animals. Although compulsive behavior is considered to characterize pathological excessive food intake, to our knowledge, no evidence has been reported of continued food seeking/intake despite its possible harmful consequences, an index of compulsive behavior. Brain monoamine transmission is considered to have a key role in vulnerability to eating disorders, and norepinephrine in medial prefrontal cortex has been shown to be critical for food-related motivated behavior.

Here, using a new paradigm of conditioned suppression, we investigated whether the ability of a foot-shock-paired conditioned stimulus to suppress chocolate-seeking behavior was reversed by previous exposure to a food restriction experience, thus modeling food seeking in spite of harmful consequences in mice. Moreover, we assessed the effects of selective norepinephrine inactivation in medial prefrontal cortex on conditioned suppression test in stressed and caloric restricted mice.

**Results:**

While Control (non food deprived) animals showed a profound conditioned suppression of chocolate seeking during presentation of conditioned stimulus, previously food restricted animals showed food seeking/intake despite its possible harmful consequences. Moreover, food seeking in spite of harmful consequences was prevented by selective norepinephrine inactivation, thus showing that prefrontal cortical norepinephrine is critical also for maladaptive food-related behavior.

**Conclusions:**

These findings indicate that adaptive food seeking/intake can be transformed into maladaptive behaviors and point to "top-down" influence on eating disturbances and to new targets for therapy of aberrant eating behaviors.

## Background

Several factors have been proposed to act on the pathogenesis of eating disorders [1,2], as individual vulnerability, stress exposure and caloric restriction [3]. Thus, animal studies have shown that chronic stress increases the consumption of "comfortable food", that is, palatable food [4], and precipitates binge eating [5]; similarly, human studies indicate that most individuals increase food intake during stress and that eating disorders usually emerge after a period of caloric restriction [6].

Compulsive food seeking characterizes some pathological overeating, as compulsive drug seeking characterizes drug-addiction [7]. Note that drugs of abuse and pathological food intake show behavioral similarities, and several brain areas, as well as neurotransmitters systems, have been involved in the reinforcement of both food and drugs, thus suggesting that natural and pharmacological stimuli activate some common neural systems [7-12]. Moreover, acute or chronic stress influences both food intake and the propensity to take drugs [10].

Recent evidence suggests the possibility of producing animal models of eating disorders [13-20]. To our knowledge, however, no evidence has been reported of continued food seeking/intake despite its possible harmful consequences, an index of compulsive behavior [21].

In this study we assessed if chronic stress is able to render palatable food seeking impervious to signals of punishment, leading to food compulsion in sated mice. Appetitive behavior for natural and drug rewards is normally suppressed by aversive stimuli or outcomes, a phenomenon called conditioned suppression [22]. Using a new conditioned suppression paradigm, we investigated whether the ability of a foot-shock-paired conditioned stimulus (CS) to suppress chocolate-seeking behavior was reversed by exposure to chronic stress, thus modeling food seeking in spite of harmful consequences in mice. Milk chocolate was chosen based on previous studies showing its rewarding properties in animals [12,23,24]. In addition, chocolate is the most commonly craved food and chocolate craving and addiction have been proposed in humans [25].

Recent evidence points to a critical role of prefrontal cortex in motivated behavior related to food or drugs, in both animals and humans [10,12,17,23,26-28]. Norepinephrine (NE) transmission in medial prefrontal cortex (mpFC) has been shown to be involved in the behavioral and central effects of drugs of abuse [29-33], and to be critical for food-related motivated behavior [12,34].

Based on these evidences, we hypothesize that norepinephrine in the mpFC has a major role also in maladaptive seeking/intake for palatable foods. Thus, we assessed if selective prefrontal NE depletion eliminates food seeking in spite of harmful consequences shown by sated mice exposed to chronic stress.

Here using a new conditioned suppression paradigm, we investigated whether the ability of a foot-shock-paired conditioned stimulus to suppress chocolate-seeking behavior was antagonized by previous exposure to a chronic stressful experience, thus modeling aberrant chocolate seeking in sated mice. Our findings demonstrate that while Control (non-food deprived) animals showed a profound conditioned suppression of chocolate seeking during presentation of conditioned stimulus, previously food deprived (FD) animals revealed a clear-cut preference for the chamber containing chocolate, thus indicating that previous exposure to a food restriction experience induces food seeking/intake despite its possible harmful consequences, which is an index of compulsive behavior. Moreover, we found that maladaptive chocolate seeking is prevented by selective norepinephrine inactivation, thus showing that prefrontal cortical norepinephrine is critical also for aberrant food-related behavior.

## Results

### Experiment 1: Conditioned Suppression Test in Control and Food Deprived groups

**Figure 1:**
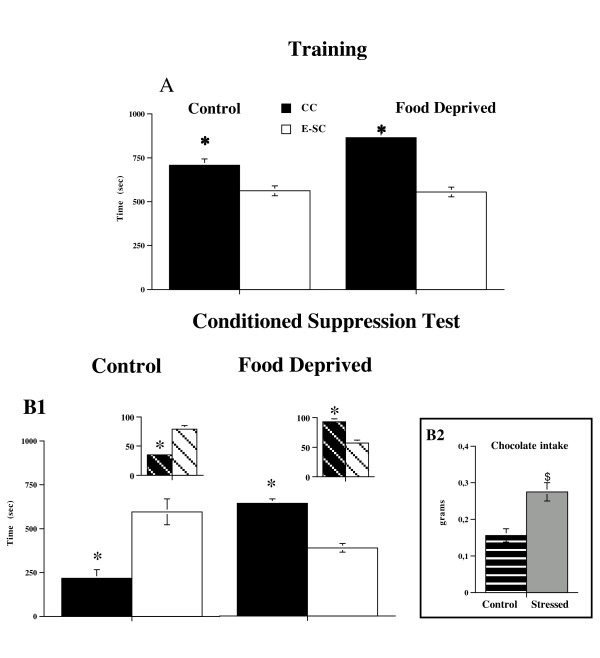
**Conditioned Suppression Test in Control and FD animals**. Panel A. Mean (sec ± SE) time spent in chamber containing chocolate (CC) and in empty-safe chamber (E-SC) during training phase. Data are expressed as mean of four days of training. Panel B. Mean (sec ± SE) time (total time) spent in chamber containing chocolate (CC) (when the aversive CS (light) was also present in the CC) and in empty-safe chamber (E-SC) during Conditioned Suppression Test (B1) by Control and FD animals. Striped pattern show partial time spent in CC and E-SC during Test by two groups; * p < 0.005 in comparison with empty-safe chamber. Chocolate intake shown by Control and FD groups on conditioned suppression test day (B2). Data are expressed as total mean grams ± SE. $ p < 0.005 in comparison with Control group.

Control (non-food deprived) and Food Deprived mice showed no significant difference in total time spent in chocolate-chamber (CC) in comparison with empty-safe chamber (E-SC) during the training phase and on the choice check test day. Comparison of time spent in chocolate-chamber and empty-safe chamber within each groups indicated that both groups showed a significant preference for the chocolate-chamber in comparison with empty-safe chamber during the training phase (Figure 1A) (Control: (F (1,14) = 20.31; p < 0.001; FD: (F (1,14) = 43.25; p < 0.0005), and on the choice check test day (Table 1) (Control: (F (1,14) = 18.21; p < 0.005; FD: (F (1,14) = 6.72; p < 0.05) aimed at assessing whether the preference for chocolate was still present the day after the conditioned stimulus (CS)-shock pairing session and before the testing session.

**Table 1:** Time (sec) spent in CC and E-SC chambers during choice check test.

	N	CC	E-SC
Control	8	698.83 ± 31.35 *	537.05 ± 21.31

FD	8	793.34 ± 93.98^&^	522.93 ± 45.28

Sham FD	8	762.99 ± 36.90^&^	506.76 ± 77.83

NE depleted FD	8	779.61 ± 60.33^&^	554.57 ± 59.16

* p < 0.005 in comparison with empty-safe chamber (E-SC); & p < 0. 05 in comparison with empty-safe chamber.

For the conditioned suppression test, statistical analysis performed on total time, revealed significant chamber × pretreatment interaction (F (2,28) = 20.23; p < 0.0005). Comparison of time spent in chocolate-chamber and empty-safe chamber within each groups showed a different behavioral pattern in the two groups. Control animalsrevealed a significant aversion for the chamber containing chocolate, in which CS was present (Figure 1B1), as shown by the time spent (sec ± SEM) in this chamber in comparison with the empty but "safe" chamber (namely, the chamber in which no conditioned threatening stimulus was present) (F (1,14) = 17.51; p < 0.001). Thus, Control animals showed a profound conditioned suppression of chocolate seeking during presentation of CS, indicating that chocolate seeking was sensitive to an adverse stimulus (Figure 1B1). By contrast, Food Deprived animals revealed a clear-cut preference for the chamber containing chocolate, as shown by the time they spent in the chamber containing chocolate compared with that in the empty but "safe" chamber (F (1,14) = 82.44; p < 0.001) (Figure 1B1). This conclusion was confirmed by statistical analysis carried out on time spent in chocolate-chamber in comparison with empty-safe chamber during presentation of the CS only (partial time) in the conditioned suppression test (F (2,28) = 27.508; p < 0.0005). While Control animals revealed a significant aversion to the chamber containing chocolate, in which CS was present (F (1,14) = 30.145; p < 0.0001), Food Deprived animals revealed a clear-cut preference for the chamber containing chocolate (F (1,14) = 23.795; p < 0.0005) during presentation of the CS only.

Moreover it's worth noting that, as expected from studies with inbred mice, the individual scores of Food Deprived animals were, for all individuals, higher than those of each mice included in Control group (Mean comparison: Chocolate-Chamber (F (1,14) = 47.44); p < 0.0005. Control = 217 ± 50; Food deprived = 577 ± 13. Empty-safe Chamber (F (1,14) = 10.038) p < 0.01. Control = 596 ± 75; Food Deprived = 349 ± 22).

### Experiment 2: Effects of selective prefrontal NE depletion on Conditioned Suppression Test

Sham Food Deprived and NE depleted Food Deprived mice showed no significant difference in total time spent in chocolate-chamber in comparison to empty-safe chamber during the training phase and on the choice check test day. Comparison of time spent in chocolate-chamber and empty-safe chamber within each groups indicated that both groups showed a significant preference for the chocolate-chamber in comparison with the empty-safe chamber during the training phase (Sham FD: (F (1,14) = 27.34; p < 0.0005; NE depleted FD: (F (1,14) = 28.71; p < 0.0005)), and on the choice check test day (Sham FD: (F (1,14) = 8.85; p < 0.05; NE depleted FD: (F (1,14) = 7.09; p < 0.05).

For the conditioned suppression test, statistical analysis performed on total time, revealed significant chamber × pretreatment interaction (F (2,28) = 3.83; p < 0.05). Comparison of time spent in chocolate-chamber and empty-safe chamber within each groups showed a different behavioral pattern in two groups.

**Figure 2:**
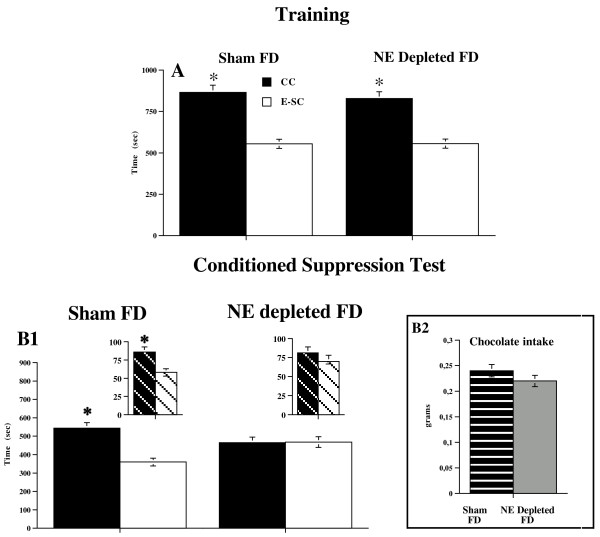
**Conditioned Suppression Test in Sham FD and NE depleted FD animals**. Panel A. Mean (sec ± SE) time spent in chamber containing chocolate (CC) and in empty-safe chamber (E-SC) during training phase. Data are expressed as mean of four days of training. Panel B. Mean (sec ± SE) time spent in chamber containing chocolate (CC) (when the aversive CS (light) was also present in the CC) and in empty-safe chamber (E-SC) in Conditioned Suppression Test (B1) by Sham FD and NE depleted FD animals. Striped pattern show partial time spent in CC and E-SC during Test by two groups; * p < 0.005 in comparison with empty-safe chamber. Chocolate intake shown by Sham FD and NE depleted FD groups on conditioned suppression test day (B2). Data are expressed as total mean grams ± SE.

Sham Food Deprived animals showed no conditioned suppression of chocolate seeking; in fact, they dramatically preferred the chamber containing food even though there was a signaled (CS, light) incoming aversive event (shock) (Figure 2B1); thus, they paralleled naïve animals (F (1,14) = 24.57; p < 0.001). On the contrary, NE depleted Food Deprived animals showed no significant preference for the chamber containing chocolate (Figure 2B1), (F (1,14) = 0.004; n.s.), thus strongly indicating that prefrontal NE depletion was responsible for maladaptive eating behavior observed in Sham Food Deprived animals.

This conclusion was confirmed by results from statistic carried out on time spent in chocolate-chamber in comparison with empty-safe chamber during presentation of the CS only (partial time) in the conditioned suppression test (F (2,28) = 11.497; p < 0.0005). While Sham Food Deprived animalsrevealed a significant preference for the chamber containing chocolate, in which CS was present (F (1,14) = 9.789; p < 0.01), NE depleted Food Deprived animals revealed no significant preference for the chamber containing chocolate (F (1,14) = 2.148; n.s.) during presentation of the CS only.

Sham and NE depleted Control mice showed no significant difference in total time spent in chocolate-chamber in comparison with empty-safe chamber during the training phase and on the choice check test day. Comparison of time spent in chocolate-chamber and empty-safe chamber within each groups showed in both groups a significant preference for the chocolate-chamber in comparison with the empty-safe chamber during the training phase (Sham Control: (F (1,14) = 19.12; p < 0.001; NE depleted Control: (F (1,14) = 16.64; p < 0.005)), and on the choice check test day (Sham Control: (F (1,14) = 15.94; p < 0.05; NE depleted Control: (F (1,14) = 13.44; p < 0.05).

**Figure 3:**
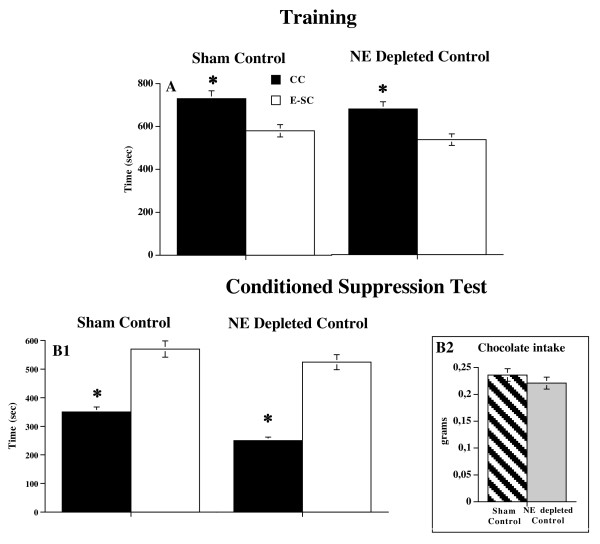
**Conditioned Suppression Test in Sham Control and NE depleted Control animals**. Panel A. Mean (sec ± SE) time spent in chamber containing chocolate (CC) and in empty-safe chamber (E-SC) during training phase. Data are expressed as mean of four days of training. Panel B. Mean (sec ± SE) time spent in chamber containing chocolate (CC) (when the aversive CS (light) was also present in the CC) and in empty-safe chamber (E-SC) in Conditioned Suppression Test (B1) by Sham Control and NE depleted Control animals; * p < 0.005 in comparison with empty-safe chamber. Chocolate intake shown by Sham Control and NE depleted Control groups on conditioned suppression test day (B2). Data are expressed as total mean grams ± SE.

For the conditioned suppression test, statistical analysis performed on total time, revealed significant chamber × pretreatment interaction (F (2,28) = 5.48; p < 0.01). Comparison of time spent in chocolate-chamber and empty-safe chamber within each groups showed in both groups a significant aversion for the chamber containing chocolate (Sham Control: (F (1,14) = 19.45; p < 0.001; NE depleted Control: (F (1,14) = 49.87; p < 0.05). (Figure 3B1), thus indicating that prefrontal NE depletion had no effect in control, non-food deprived, animals.

### Chocolate intake and body weight

Regarding experiment 1, statistical analysis carried out on chocolate intake data during the training phase of the conditioned suppression test in Control and Food Deprived animals revealed a significant pretreatment (F (1,42) = 166.43; p < 0.0005) and day effect (F (3,42) = 8.78; p < 0.0005) effect. Simple effect analyses revealed a significant effect of day for both groups (Control: (F (3,28) = 4.667; p < 0.01; Food Deprived: F (3,28) = 6.365; p < 0.01) and a significant difference between Control and Food Deprived at all time points, thus indicating that both groups showed a daily increase of chocolate intake through the training phase and that Food Deprived animals ate more chocolate than Control animals.

Regarding chocolate intake on the choice check and conditioned suppression test days, statistical analysis revealed a significant difference in chocolate consumption between Control and Food Deprived animals (choice check test: (F (1,14) = 82.89; p < 0.0005; suppression test day: (F (1,14) = 15.13; p < 0.005), indicating a compulsive chocolate intake in Food Deprived animals.

Results from chocolate intake during conditioned suppression test day show thatprevious exposure to a stressful food restriction experience induces food seeking/intake despite its possible harmful consequences (Figure 1B2), which is an index of compulsive behavior.

Moreover, it should be noted that the behavior shown by the Food Deprived mice cannot be ascribed to a homeostatic response to dietary deficiencies because they returned to *ad libitum *feeding and to initial body weight (F (1,14) = 0.033; n.s.) before Conditioned Suppression test day and no significant difference in body weight (F (1,14) = 3.17; n.s.) (Table 2) was evident between Control and Food Deprived groups on the Conditioned Suppression test day. Thus, these data indicate that food seeking in spite of harmful consequences observed in Food Deprived mice was not determined by general motivation to eat, akin to hunger, but rather by a more specific motivational state, akin to craving.

**Table 2:** Initial body weight (before food restriction) and body weight on conditioned suppression test day

	N	Initial Body Weight	CS test day Body Weight
Control	8	24.41 ± 0.31	25.75 ± 0.22

FD	8	25.08 ± 0.41	25.16 ± 0.25

Sham FD	8	23.53 ± 0.48	23.96 ± 0.61

NE depleted FD	8	24.76 ± 0.83	25.45 ± 0.73

Regarding experiment 2, statistical analysis carried out on chocolate intake data in both Sham Food Deprived and NE Depleted Food Deprived animals during the training phase of the conditioned suppression test revealed a significant day effect only (F (3,42) = 17.53; p < 0.0005). Simple effect analyses revealed a significant effect of day for both groups (Sham FD: (F (3,28) = 8.266; p < 0.001; NE Depleted FD: (F (3,28) = 7.323; p < 0.001), thus indicating that all groups showed daily increased chocolate intake through the training phase.

Concerning chocolate intake on the choice check and conditioned suppression test days in Food Deprived animals, statistical analysis revealed no significant differences in chocolate consumption between Sham Food Deprived and NE depleted Food Deprived groups (choice check test: F (1,14) = 0.21 n.s.; suppression test: F (1,14) = 9.85; n.s.) (Figure 2B2), thus ruling out that the effects of prefrontal NE depletion on the conditioned suppression test can be ascribed to the different consumption pattern shown by NE depleted Food Deprived and Sham Food Deprived animals. Finally, statistical analysis for chocolate intake on the choice check and conditioned suppression test days in Control, non-food deprived animals revealed no significant differences in chocolate consumption between Sham control and NE depleted control groups (choice check test: F (1,14) = 0.017 n.s.; suppression test: F (1,14) = 0.086; n.s.) (Figure 3B2).

Regarding weight results from conditioned suppression experiments, a statistical analysis revealed no significant difference on the day the experiment started (that is before food restriction schedule started) between Control and Food Deprived animals (F (1,14) = 1.658; n.s.) or between Sham Food Deprived and NE depleted Food Deprived mice (F (1,14) = 1.66; n.s.) and no significant difference on the conditioned suppression test day between Control and Food Deprived animals (F (1,14) = 3.17); n.s.) or between Sham Food Deprived and NE depleted Food Deprived mice (F (1,14) = 1.52; n.s.) (Table 2). Finally, although a significant difference was observed between Control and Food Deprived groups (F (1,14) = 154.57): p < 0.001), there was no significant difference between Sham Food Deprived and NE depleted mice Food Deprived (F (1,14) = 1.52; n.s.) on the choice check test day. A comparison between initial weight and weight on the conditioned suppression test day, carried out for food restricted groups (Food deprived, Sham Food deprived, NE depleted Food deprived), revealed no significant effect for Food Deprived (F (1,14) = 0.03; n.s.), Sham Food Deprived (F (1,14) = 0.31; n.s.) or NE depleted Food Deprived (F (1,14) = 0.38; n.s.) groups.

Thus, the different behavioral outcomes observed in Sham Food Deprived and NE depleted Food Deprived groups cannot be ascribed to a difference in consumption patterns during the training phase of the conditioned suppression test or to unspecific effects of prefrontal NE depletion on recovery of body weight when animals returned to *ad libitum *feeding. In fact, no significant difference in chocolate consumption was found between Sham Food Deprived and NE depleted Food Deprived groups on the training days (F (3,42) = 0.916; n.s.), the choice check (F (1,14) = 0.21; n.s.) or the test (F (1,14) = 9.85; n.s.) (Figure 2B2) days, and no significant difference in body weight was observed between Sham Food Deprived and NE depleted Food Deprived groups on the choice check (F (1,14) = 1.52; n.s.) or the conditioned suppression test (F (1,14) = 2.46; n.s.) days (Table 2).

### Conditioned Aversion Test

**Figure 4:**
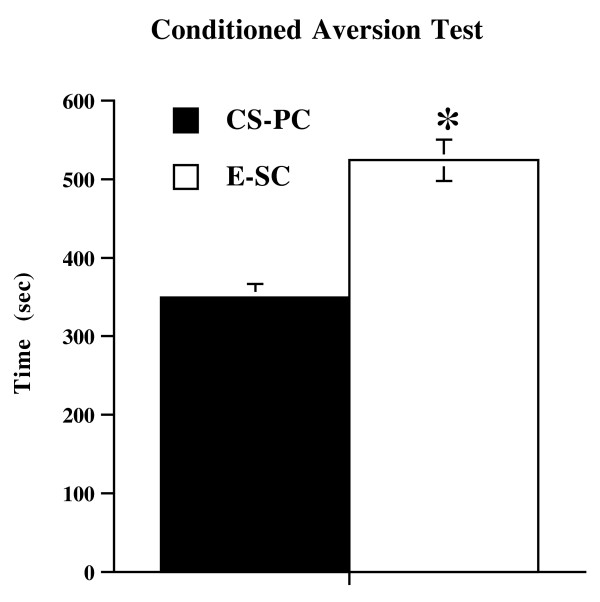
**Conditioned Avoidance Test in FD animals**. Mean (sec ± SE) time spent in the conditioned stimulus-paired chamber (CS-PC) and in empty-safe chamber (E-SC) by FD animals; * p < 0.05 in comparison with CS-PC.

Data from the conditioned aversion test in Food Deprived animals (additional conditioned avoidance experiment) revealed a significant chamber effect (F (2,21) = 7.97; p < 0.005). Food Deprived animals showed a significant aversion for the conditioned stimulus-paired chamber (CS-PC) in comparison with the empty-safe chamber (E-SC) (F (1,14) = 12.17; p < 0.005) (Figure 4). Thus, the effects shown by Food Deprived mice in the conditioned suppression test cannot be ascribed to unspecific impairment of the foot-shock-CS association induced by food restriction, because Food Deprived animals was able to associate the shock with the light.

**Figure 5:**
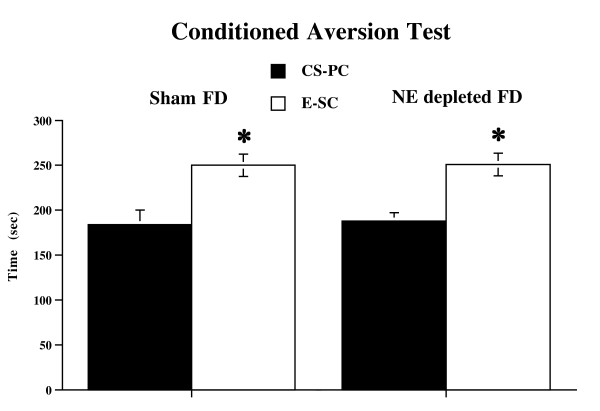
**Conditioned Avoidance Test in Sham FD and NE depleted FD animals**. Mean (sec ± SE) time spent in the conditioned stimulus-paired chamber (CS-PC) and in empty-safe chamber (E-SC) by Sham and NE depleted animals; * p < 0.05 in comparison with CS-PC.

Data from the conditioned aversion test in Sham Food Deprived and NE depleted Food Deprived groups revealed a significant chamber effect (F (1,14) = 12.38; p < 0.0005) and no significant difference between Sham Food Deprived and NE depleted Food Deprived groups. Sham Food Deprived and NE depleted Food Deprived groups showed a clear aversion to the conditioned stimulus-paired chamber in comparison with empty-safe chamber (Sham FD: (F (1,14) = 6.60; p < 0.05; NE depleted FD: (F (1,14) = 9.41; p < 0.01) (Figure 5). Thus, the effects of prefrontal NE depletion in the conditioned suppression test cannot be ascribed to unspecific impairment of the foot-shock-CS association because there were no significant differences between Sham Food Deprived and NE depleted Food Deprived mice in the conditioned avoidance test, in agreement with previous results showing that prefrontal NE depletion did not interfere with either associative or mnemonic processes.

### Shock Sensitivity

**Figure 6:**
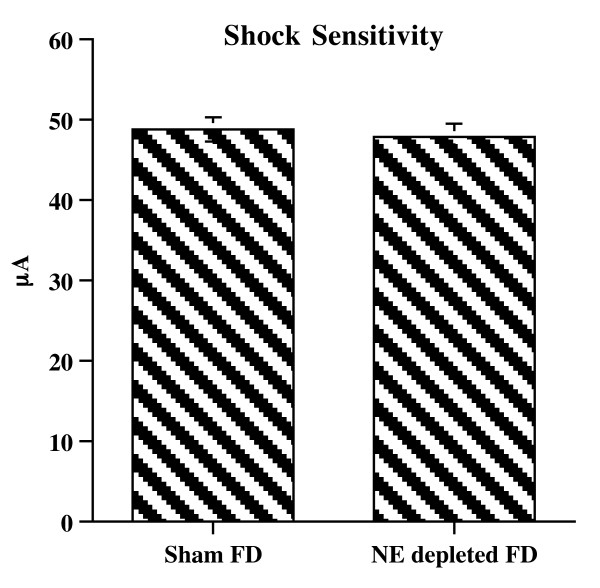
**Shock sensitivity in Sham FD and NE Depleted FD animals**. Mean (μA ± SE) shock threshold observed in Sham FD and NE Depleted FD animals.

Statistical analysis revealed no significant difference between Sham Food Deprived and NE depleted Food Deprived groups (F (1,14 = 0.17; n.s.). Performances of the two groups cannot be ascribed to differences in shock sensitivity, because similar mean shock thresholds (μA) were observed in Sham FD and NE depleted FD mice (Sham FD = 48.8 ± 1.6: NE depleted FD = 48.0 ± 1.7) (Figure 6).

### Selective NE depletion in medial prefrontal cortex

Statistical analysis for the effects of prefrontal NE depletion on dopamine and NE tissue levels in the mpFC showed a significant effect only for NE (F (1,14) = 228.3; p < 0.0005). Selective depletion of NE prefrontal cortical afferents produced an approximately 90% decrease in NE tissue levels (Sham = 708 ± 39; NE depleted = 68 ± 19 ng/g wet tissue), whereas it spared DA tissue levels (Sham = 221 ± 18; NE depleted = 214 ± 17 ng/g wet tissue). No significant effect was evident for either NE or dopamine in the nucleus accumbens (NE: Sham = 715 ± 35; NE depleted = 767 ± 39; Dopamine: Sham = 12899 ± 735; NE depleted = 12767 ± 815 ng/g wet tissue).

Note that NE depletion method used here does not affect either other brain areas such as cingulated cortex as previously reported [32].

## Discussion

Here, using a new conditioned suppression paradigm, we report evidence of food seeking/intake in the face of adverse consequences. We modeled this maladaptive eating behavior in sated micepreviously exposed to a food restriction experience.

In fact, in the conditioned suppression test Control (non-food deprived) animals(experiment 1) revealed a significant aversion for the chamber containing chocolate, in which CS was present (that is, the stimulus previously paired with foot-shock), as shown by the time spent) in this chamber in comparison with the empty but "safe" chamber (namely, the chamber in which no conditioned threatening stimulus was present), thus, indicating that chocolate seeking was sensitive to an adverse stimulus. By contrast, Food Deprived animals revealed a clear-cut preference for the chamber containing chocolate, thus indicating that previous exposure to a food restriction experience induces food seeking/intake despite its possible harmful consequences, which is an index of compulsive behavior [22].

Moreover Food Deprived animals, although sated, ate more chocolate than Control animals on the conditioned suppression test day thus indicating an excessive chocolate intake. Note that the behavior shown by the Food Deprived mice cannot be ascribed to a homeostatic response to dietary deficiencies because they returned to *ad libitum *feeding and to initial body weight before the Conditioned Suppression test day and no significant difference in body weight was evident between Control and Food Deprived groups on the Conditioned Suppression test day. Thus, these data indicate that excessive chocolate seeking observed in Food Deprived mice was not determined by general motivation to eat, akin to hunger, but rather by a more specific motivational state, akin to craving.

**Figure 7:**
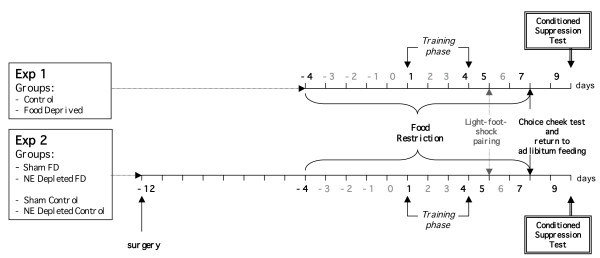
**Schematic time-line of the experimental procedures (experiment 1 and experiment 2)**.

However, since food deprived mice were exposed to chocolate in the test apparatus while being food deprived (see experimental procedures, Figure 7), chocolate might be more rewarding in the food deprived than in the control mice, thus making the food deprived mice more motivated to consume chocolate during the final test. Further experiments will be carried out in order to assess this point.

Moreover, conditioned suppression in previously food-restricted animals may involve an incentive learning process [35] that allows the animals to assign an appropriate value to a reward that is modulated by its motivational states. This learning process is engaged when animals contact and experience the reward in the relevant state. Thus, exposure to chocolate during training, that is when animals are still in food-restriction, may have increased the perceived salience of chocolate due to the motivational state induced by feeding regimen that would lead to an increased value of the reinforce at the moment of test, that is when animals are yet in free-feeding for two days. This is consistent with results of Balleine and Dickinson (1998) [35] showing that the deprived state able to increase incentive value is critical if animals are exposed far from testing but not at the test.

Note that we aimed at increasing the salience and the motivational value of chocolate before testing, by modulating the motivational state by food-restriction, an experience that makes animals deprived and that is, at the same time, able to sensitize the brain reward system [36-39].

The effects shown by Food Deprived mice in the conditioned suppression test cannot be ascribed to unspecific impairment of the foot-shock-CS association induced by food restriction, because Food Deprived animals were able to associate the shock with the light (that is, the conditioned stimulus used in the conditioned suppression experiment), as shown by an additional conditioned avoidance experiment.

Food restriction in rodents is commonly considered a stressful conditions leading to, among other effects, altered sensitization of brain reward systems [36-39]. Accordingly, a number of reports in the last two decades have shown that stress hormones as glucocorticoids, ACTH or CRF are affected by food restriction also depending on the circadian rhythm [40-44]. Although we have not assessed hormones in our experimental groups, it is very likely that the food-restriction paradigm we used affects hormones consistently with what observed in the literature.

Because the behavioral outcome shown in conditioned suppression test is reminiscent of aberrant eating behaviors observed in humans, the animal model proposed here could be a good tool for understanding how maladaptive environmental influences, such as exposure to chronic stress, previous caloric restriction and access to highly palatable food, contribute to aberrant eating behaviors, as obesity. Obesity is a multifactorial disease caused by environmental and genetic factors and by the complex interactions among them [3,10]. Of the environmental factors that influence obesity, the availability of seductive foods is the most obvious. Moreover, stress has a potential role in obesity [4,10]. As previously underlined, food restriction in rodents is commonly considered a stressful condition leading to altered sensitization of brain reward systems [36-39] and greater sensitization of the reward system can lead to excessive intake of highly palatable food [6]. In fact, repeated stimulation of reward pathways through highly palatable food may lead to neurobiological adaptations that make the ingestive behavior more compulsive [10].

Besides environmental also genetic factors play a large role in obesity [3,10]. Genetic studies have revealed point mutations that are of importance for obesity and genetic predisposition can be important in determining the degree of obesity in response to high-fat diet [45]. Finally, studies in mice show that certain strains of mice, as C57BL/6, have increased susceptibility to weight gain on a high-fat diet, showing genetic predisposition to obesity [45].

Based on evidence pointing to the involvement of prefrontal cortical NE transmission in the behavioral and central effects of drugs of abuse [29-33], and in food-related motivated behavior (12,34), we tested the hypothesis that NE in mpFC has a major role also in aberrant motivation related to seeking of palatable foods, assessing the effects of selective prefrontal NE depletion on conditioned suppression test.

While Sham Food Deprived animals showed no conditioned suppression of chocolate seeking thus paralleling naïve animals, NE depleted Food Deprived animals showed no significant preference for the chamber containing chocolate, thus strongly indicating that prefrontal NE transmission was responsible for maladaptive eating behavior observed in Sham Food Deprived animals.

These results extend previous findings on the role of prefrontal NE transmission in food-related motivated behavior [11,12] and show for the first time that prefrontal cortical NE has a major role also in aberrant behaviors related to seeking/intake of palatable foods. Moreover, they are consistent with a critical role of prefrontal cortical NE in the attribution of motivational salience [34] and suggest strongly that food seeking in spite of harmful consequences is the expression of aberrant motivation controlled by noradrenergic transmission in the mpFC.

It must be taken into account, however, that prefrontal NE depletion did not reinstate conditioned avoidance in food deprived mice, since depleted mice showed neither preference nor aversion of the chocolate-chamber. Therefore, although prefrontal NE plays an important role in the insensitivity to presentation of an aversive CS in food-deprived animals, it is unlikely the sole responsible mediator of this effect.

Recent evidence points to a critical role of prefrontal NE transmission in food-related motivated behavior through modulation of dopamine in nucleus accumbens [12]. As dopaminergic transmission in nucleus accumbens is the most clearly established mechanism reinforcing the effects of rewarding food [10,19], the influence of prefrontal NE depletion in conditioned suppression test, observed in the present experiments, could depend on the impaired response of prefrontal-accumbal catecholamine system [12] to highly motivating stimuli [34].

In fact, NE transmission is necessary for motivational salience attribution to reward related stimuli through dopaminergic transmission within nucleus accumbens that is considered to mediate the hedonic impact of reward or some aspects of reward learning [46]. Moreover, prefrontal NE is crucial for motivational salience related to stimuli perceived as provided of intense salience by the individual organism depending on either their intrinsic properties (intense or mildly salient) or the motivational state of the organism, in the latter case affecting "perceived" salience. Food Deprived mice are likely to be characterized by sensitized reward system that leads to perceive palatable food as highly salient due not only to its intrinsic properties but also to the motivational state of the organism. This makes chocolate being provided of intense motivational salience that produces disruption of conditioned suppression observed in our experiments. Noradrenergic prefrontal transmission, probably through modulation of dopamine release within the nucleus accumbens, is a necessary condition for motivational processing of reward related stimuli. This is the starting point to possible feeding behavior driven by aberrant motivation and leading to excessive palatable food intake.

Further experiments will be carried out in order to assess this point.

Finally, a role of Corticotropin Releasing Factor (CRF) may be also envisaged due to its role in the possible stressful effects of food-restriction on nucleus accumbens either, directly or through prefrontal cortical NE. CRF has been shown to amplify positive motivation to rewards due to its action in nucleus accumbens shell [47]. Moreover, CRF is known to control locus coeruleus mediated activation of NE in the prefrontal cortex [48] that, as we have shown, controls accumbal dopamine response to natural (e.g. chocolate) or pharmacological rewards [12]. Such an action of prefrontal cortical NE on dopamine transmission in the accumbens is likely to involve alpha-1 receptors as a pioneering study on prefrontal NE regulation of the accumbal dopamine response to amphetamine has shown [29].

Note that the effects of prefrontal NE depletion in the conditioned suppression test cannot be ascribed to unspecific impairment of the foot-shock-CS association because there were no significant differences between Sham Food Deprived and NE depleted Food Deprived mice in the conditioned avoidance test, in agreement with previous results showing that prefrontal NE depletion did not interfere with either associative or mnemonic processes [32,33]. Moreover, the shock sensitivity of Sham Food Deprived and NE depleted Food Deprived mice was not significantly different. In addition, data from Sham Control and NE depleted Control groups clearly indicate that prefrontal NE depletion has no significant effect in non-food restricted animals. In fact, prefrontal NE depletion did not abolish the aversion for the chocolate-chamber showed by Sham Control animals.

Finally, the different behavioral outcomes observed in Sham Food Deprived and NE depleted Food Deprived groups cannot be ascribed to a difference in consumption patterns during the training phase of the conditioned suppression test or to unspecific effects of prefrontal NE depletion on recovery of body weight when animals returned to *ad libitum *feeding. In fact, no significant difference in chocolate consumption was found between Sham Food Deprived and NE depleted Food Deprived groups on the training days, the choice check or the test days, and no significant difference in body weight was observed between Sham Food Deprived and NE depleted Food Deprived groups on the choice check or the conditioned suppression test.

Drug abuse and palatable food intake show behavioral similarities, and several brain areas, as well as neurotransmitter systems, have been involved in the reinforcement of both food and drugs, thus suggesting that natural and pharmacological stimuli activate some common neural systems [7-12,23,26,31,49]. Because a hallmark feature of addiction is compulsive drug use in the face of adverse consequences [21,22], our results might not only be helpful in the search for neurobiological substrates of aberrant eating behaviors, but might also provide insight into the neural mechanisms of drug addiction. Finally, the model proposed can be helpful to envisage new therapeutic targets and develop suitable strategies for aberrant eating behaviors.

## Conclusions

Here we report evidence of palatable food seeking/intake in the face of adverse consequences. We modeled this behavior in sated mice exposed to a food restriction experience and demonstrate that food seeking in spite of harmful consequences is prevented by selective NE inactivation in medial prefrontal cortex.

Using a conditioned suppression paradigm, we investigated whether the ability of a foot-shock-paired conditioned stimulus to suppress chocolate-seeking behavior was antagonized by previous exposure to a food restriction experience, thus modeling aberrant chocolate seeking in mice. Control animals showed a profound conditioned suppression of chocolate seeking during presentation of conditioned stimulus, indicating that chocolate seeking was sensitive to an adverse stimulus. By contrast, Food Deprived animals showed a maladaptive chocolate seeking/intake, thus indicating that previous exposure to a food restriction experience induces food seeking/intake despite its possible harmful consequences, which is an index of compulsive behavior.

The behavioral outcome shown in conditioned suppression test is reminiscent of aberrant eating behaviors in humans, such as obesity and binge-eating, that can be driven by environmental factors apart from metabolic control. Our results show, for the first time, the possibility of modeling in sated mice aberrant palatable food seeking/intake in the face of adverse consequences, a major characteristic of addiction.

The animal model proposed here could be a good tool for understanding how maladaptive environmental influences, such as exposure to chronic stress and access to highly palatable food, contribute to aberrant eating behaviors.

Moreover, we found that food seeking in spite of harmful consequences is prevented by selective NE inactivation in mpFC, thus demonstrating that NE transmission in mpFC is critical for food-related motivated behavior. These results also extend previous findings on the role of prefrontal NE transmission in food-related motivated behavior by showing that prefrontal cortical NE has a major role also in maladaptive eating behaviors related to seeking/intake of palatable foods, thus pointing to "top-down" influence on eating disturbances and to new targets for therapy of aberrant eating behaviors.

## Methods

All experiments were conducted in accordance with the Italian national law (Decreto Legislativo no. 116, 1992) governing the use of animals for research (permit by Ministero della Salute, reference no. 149/2005-B, 04/11/2005).

### Animals

Male mice of the inbred C57BL/6JIco (C57) strain (Charles River, Como, Italy), which are commonly used in neurobehavioral phenotyping, 8-9 weeks old at the time of the experiments, were housed as previously described and maintained in a 12 hr/12 hr light/dark cycle (light on between 07.00 a.m. and 07.00 p.m.) [12,32,33]. Each experimental group was comprised of 8 animals.

### Drugs

Chloral hydrate, 6-hydroxydopamine (6-OHDA) and GBR 12909 (GBR) were purchased from Sigma (Sigma Aldrich, Milan, Italy). Chloral hydrate (350-450 mg/kg) and GBR (15 mg/Kg) were dissolved in saline (0.9% NaCl) and injected intraperitoneally (i.p.) in a volume of 10 ml/kg. 6-OHDA was dissolved in saline containing Na-metabisulphite (0.1 M).

### Selective NE depletion in medial prefrontal cortex

Anesthesia and surgical set were carried out as previously described [12,32-34]. Animals were injected with GBR (15 mg/Kg) 30 min before the 6-OHDA microinjection in order to protect dopaminergic neurons. Bilateral injection of 6-OHDA (1.5 μg/0.1 μl/2 min for each side) was made into the mpFC (coordinates: +2.52 AP; ± 0.6 L; - 2.0 V with respect to bregma [50] through a stainless steel cannula (0.15 mm outer diameter, UNIMED, Switzerland) connected to a 1 μl syringe by a polyethylene tube and driven by a CMA/100 pump (NE depleted group). The cannula was left in place for an additional 2 min after the end of the infusion. Sham animals were subjected to the same treatment, but received intracerebral vehicle after GBR administration. Note that in previous experiments no significant difference was observed between Sham-treated and naïve animals in basal or pharmacological/natural stimuli-induced prefrontal NE or dopamine outflow or in place conditioning test [12,32-34,51], thus ruling out the action of GBR on the observed effects in the present experiments.

Animals were used for behavioral experiments 7 days after surgery. NE and dopamine tissue levels in the mpFC were assessed by HPLC-EC analysis, as previously described [12,32-34], to evaluate the extent of depletion.

### Food Restriction

As food restriction is a stressful experience [37,52], in the chronic stressful condition the animals were placed on a moderate food-restriction schedule [51,54] 5 days before conditioned suppression experiments started. Mice were assigned to a feeding regimen: they either received food *ad libitum *or were subjected to a food-restricted regimen (FR). In the food restricted condition, food was delivered once daily (07.00 p.m.) in a quantity adjusted to induce a loss of 15% of the original body weight. In the control condition, food was given once daily (07.00 p.m.) in a quantity adjusted to exceed daily consumption.

Two days before testing (day 7 of training procedure) animals were returned to *ad libitum *feeding to rule out any effects of dietary deficiencies on the conditioned suppression test day (day 9). Thus, all testing sessions were carried out at least 48 hours after food was again available *ad libitum *and animals reached their body weight before restriction.

### Conditioned suppression test

The apparatus used for the conditioned suppression test was a modified version of a place conditioning apparatus [12,32-34,37]; it consisted of two gray Plexiglas chambers (15 × 15 × 20 cm) and a central alley (15 × 5 × 20 cm) with a stainless steel grid floor. A halogen lamp (10 W, Lexman) was located under the grid floor. Two sliding doors (4 × 20 cm) connected the alley to the chambers. In each chamber two triangular parallelepipeds (5 × 5 × 20 cm) made of black Plexiglas and arranged in different pattern (always covering the same surface of the chamber) were placed to make much easier for the animals to distinguish the two chambers. A Plexiglas cup (3.8 cm diameter) was placed in each chamber: in one, the cup contained 1 g of milk chocolate (Kraft); in the other, the cup was empty. The procedure was as follows: from day 1 to day 4 (training phase) mice were placed individually in the alley; the sliding doors were opened to allow them to enter freely in both chambers and to explore the entire apparatus for 30 minutes. The time spent (sec ± SEM) in each of the two chambers (i.e., the one with the cup containing chocolate and the one with the empty cup) and in the center was recorded throughout. The choice of the chamber containing chocolate was assessed by the time spent in it. On day 5, animals were exposed to light-foot-shock pairings. Acquisition of the conditioned stimulus CS (light)-shock association was established in a different apparatus comprised of one, 15 × 15 × 20 cm Plexiglas chamber with a black and white striped pattern on two walls (to make it very different from the conditioned suppression apparatus) and with a stainless steel grid floor through which the shocks were delivered. The light was produced by a halogen lamp (10 W, Lexman), located under the grid floor, that was turned on for five, 20-sec periods every 100 sec.; in each period, after the light had been on for 19 sec a 1 sec 0.15 mA scrambled foot-shock was delivered. This session of light-shock association lasted 10 min and was followed by a 10 min rest period and then by another, identical 10 min light-shock association session; overall, the mice received ten light-foot-shock pairings in a 30 min session.

On day 6, the mice were left undisturbed in their home cage. On day 7, the animals were subjected to the same procedure as in the training phase to evaluate whether the previous light-foot-shock pairings would affect, in an unspecific way, the choice of the chamber containing chocolate (Choice Check Test). Then, they were returned to *ad libitum *feeding to rule out any effects of dietary deficiencies on the conditioned suppression test day. On day 8, the mice were left undisturbed in their home cage. Finally, on day 9 the conditioned suppression of chocolate seeking was assessed in a test session that lasted 20 min in which the mice had access to chocolate in one of the two chambers in which chocolate had been placed during the training phase. In the chamber containing chocolate, CS (light) was presented according to the paradigm used for the light-foot-shock association (except for the 10 min rest period, which was discontinued). The light was produced by a halogen lamp, located under the grid floor that was turned on for 20-sec periods every 100 sec. This session lasted 20 min; overall, the mice received ten, 20-sec periods in a 20 min session.

The testing session began with the first 20-sec period of light. The time spent in each of the two chambers, the one containing chocolate and the other empty but "safe" one (the chamber in which no conditioned threatening stimulus was present), was recorded throughout the session.

All experiments were carried out in experimental sound-attenuated rooms indirectly lit by a standard lamp (60-W). For all behavioral tests, data were collected and analyzed by the 'EthoVision' (Noldus, The Netherlands), a fully automated video-tracking system [55]. The acquired digital signal was then processed by the software to extract "time spent" (in seconds) in the chambers, which was used as raw data for preference/aversion scores in each sector of the apparatus for each subject.

### Experiment 1: Conditioned Suppression Test in Control and Food deprived groups

For the first experiment two groups of mice, Control and Food Deprived, were used. Food Deprived mice were placed on a moderate food-restriction schedule 5 days before the test began (from day -4 to day 7); this schedule was maintained until 48 hours before the conditioned suppression test. From day 1 to day 4, Control (non-food deprived) and Food deprived mice were subjected to training phase. On day 5, animals were exposed to light-foot-shock pairing. On day 6, the mice were left undisturbed in their home cage. On day 7, the animals were subjected to Chocie Check Test and then Food Deprived animals were returned to *ad libitum *feeding. On day 8, the mice were left undisturbed in their home cage. Finally, on day 9 animals were subjected to Conditioned Suppression Test (Figure 7).

### Experiment 2: Effects of selective prefrontal NE depletion on Conditioned Suppression Test

To test the hypothesis that prefrontal cortical NE has a major role also in aberrant motivation related to seeking/intake of palatable foods, we investigated effects of selective prefrontal NE depletion on conditioned suppression test.

Two groups of food deprived (FD) mice, Sham (Sham FD) and NE depleted (NE depleted FD), were used. Both groups were placed on a moderate food-restriction schedule 5 days before the test started (from day -4 to day 7); this schedule was maintained until 48 hours before the conditioned suppression test.

Moreover, other two groups of animals were used to evaluate the effects of prefrontal NE depletion in control (non-food deprived) animals: Sham Control and NE depleted Control.

Before the training phase started mice were randomly assigned to one of the two groups (Sham, NE depleted) and subjected to surgery. Both Control Deprived and Food Deprived groups were subjected to surgery and after seven days they were used for behavioral test. From day -4 to 7 day, Food Deprived mice (Sham, NE depleted) were subjected to food restriction procedure. From day 1 to day 4 both Control and Food Deprived groups were subjected to training phase. On day 5, animals were exposed to light-foot-shock pairing. On day 6, the mice were left undisturbed in their home cage. On day 7, the animals were subjected to Chocie Check Test and then Food Deprived groups were returned to *ad libitum *feeding. On day 8, the mice were left undisturbed in their home cage. Finally, on day 9 animals were subjected to Conditioned Suppression Test (Figure 7).

### Conditioned Avoidance Test

One group of Food Deprived mice was tested in a conditioned avoidance test to rule out any unspecific effects of food restriction on light-shock association ("additional conditioned avoidance experiment"). The conditioned avoidance test was conducted like the conditioned suppression test, but there was no chocolate in either of two chambers.

One week after the conditioned suppression test, Sham Food Deprived and NE depleted Food Deprived groups were subjected to another light-foot-shock association session; four days later, they were tested in a ten-minute conditioned avoidance test to rule out any unspecific effects of prefrontal NE depletion.

### Shock sensitivity

To rule out alterations in sensitivity to foot-shock with NE depletion, Sham Food Deprived and NE depleted Food Deprived mice were tested for foot-shock sensitivity. The latter was measured using a modified version of the flinch-jump test [55]. Individual mice were placed in the testing apparatus for a 1-2 min acclimation period; no background noise was presented during the testing period. Then, they received 6 series of 6 shocks (1 sec), ranging from 15 to 150 μA, delivered at 20 sec intervals through the grid floor. The series of shocks were delivered in alternating ascending and descending order; the first series was in ascending order. Shock threshold was defined as the lowest shock intensity (μA) at which an animal's hind foot left the grid floor. For each mouse, the mean value of shock thresholds recorded in each series was calculated.

### Chocolate consumption and body weight

Chocolate consumption was assessed during the conditioned suppression experiments. Intake was evaluated by weighing leftover chocolate at the end of each training phase session (on days 1-4), on day 7 (choice check test) and on day 9 (conditioned suppression test). Finally, mice were weighed daily throughout the experiments.

### Statistics

The effects of prefrontal NE depletion on tissue levels of dopamine and NE in the mpFC and nucleus accumbens were analyzed by one-way ANOVA (Sham, NE depleted).

Concerning Conditioned suppression test, statistical analyses were performed by calculating the time (sec) spent (total time) in the center (Center, CT), in the chamber containing chocolate (Chocolate-Chamber, CC) and in the empty-safe chamber (Empty-Safe Chamber, E-SC) in the training phase (overall mean of four days of training) and on the choice check and conditioned suppression test days. Moreover, the time (sec) spent in CT, CC and E-SC during presentation of the conditioned stimulus only (partial time) was also analyzed in the conditioned suppression test. Data were analyzed using repeated-measures ANOVA, with one between factor (pretreatment, two levels: Control, Food Deprived or Sham, NE depleted) and one within factor (chamber, three levels: CT, CC, E-SC). Moreover, as the important comparisons are those between the CC and E-SC chambers, mean comparisons of time spent in CC and E-SC chambers were made using repeated-measure ANOVA within each group.

Data from the conditioned aversion test of Food Deprived animals (additional conditioned avoidance experiment) were analyzed using repeated-measures ANOVA (chamber, three levels: CT, conditioned stimulus-paired chamber (CS-PC) E-SC). Data from the conditioned aversion test of Sham and NE depleted were analyzed using repeated-measures ANOVA, with one between factor (pretreatment, two levels: Sham, NE depleted) and one within factor (chamber, three levels: CT, CS-PC, E-SC). Moreover, as the important comparisons are those between the CS-PC and E-SC chambers, mean comparisons of time spent in CS-PC and E-SC chambers were made using repeated-measures ANOVA within each group.

Concerning shock sensitivity, statistical analyses were performed using one-way ANOVA, (Sham, NE depleted).

Chocolate intake during the training phase of the conditioned suppression test was analyzed using repeated-measures ANOVA with one between factor (pretreatment, two levels: Control, Food Deprived; or Sham, NE depleted) and one within factor (day, four levels: d1, d2, d3, d4). Simple effects were assessed by one-way ANOVA for each time point. Moreover, within each group repeated-measures ANOVA (day, four levels: d1, d2, d3, d4) on chocolate intake were performed.

Chocolate intake on the choice check and conditioned suppression test days was analyzed using one-way ANOVA.

Finally, animals' weight was also recorded. Mice were weighed on the first day of the experiment (before food restriction schedule started) and on the choice check test and conditioned suppression test days. Data were analyzed by one-way ANOVA for each experiment (Control vs. Food Deprived; or Sham vs. NE depleted). Moreover, for experiments with food-restricted animals (Food Deprived, Sham Food Deprived, NE depleted Food Deprived groups), initial weight and weight on conditioned suppression test day was compared by repeated-measures ANOVA.

## Abbreviations

CC: chocolate-chamber; CS: conditioned stimulus; CS-PC: conditioned stimulus-paired chamber; CT: center; ES-C: empty-safe chamber; FD: food deprived; FR: food restriction; GBR: GBR 12909; mpFC: medial prefrontal cortex; NE: norepinephrine; 6-OHDA: 6-hydroxydopamine.

## Authors' contributions

RV, ECL and SP-A designed research and analyzed data. RV and SP-A wrote the paper. ECL, EP and RV performed experiments. All authors discussed the results and commented on the manuscript, and read and approved the final manuscript. The authors declare no conflict of interest.
